# Role Analysis of the *scarb1* Gene in the Pigmentation of *Neocaridina denticulata sinensis*

**DOI:** 10.3390/ani15070901

**Published:** 2025-03-21

**Authors:** Lili Zhang, Guodong Wang, Haifan Li, Tanjun Zhao

**Affiliations:** 1 Key Laboratory of Healthy Mariculture for the East China Sea, Ministry of Agriculture and Rural Affairs, Fisheries College, Jimei University, Xiamen 361021, China; llzhang@jmu.edu.cn (L.Z.); 19396365986@163.com (H.L.); zhaotanjun233@163.com (T.Z.); 2 State Key Laboratory of Mariculture Breeding, Fisheries College, Jimei University, Xiamen 361021, China

**Keywords:** scavenger receptor class B type I, carotenoid, RNAi, SNP genotyping

## Abstract

Body color is a key economic trait for *Neocaridina denticulata sinensis*, an important ornamental shrimp. *Scarb1* may be an important mediator of astaxanthin uptake, changing the shrimp’s body color. To discover the relationship between *scarb1* and the pigmentation of cherry shrimp, the expression profiles, RNAi, and SNP genotyping of *scarb1* were studied. There were significant differences in four color populations and five development stages (*p* < 0.05). The highest expression level of *scarb1* appeared in the red population and the pre-nauplius stage. Exposure to *scarb1* dsRNA increased the number and development of chromatophores at the metanauplius stage, but almost no phenotypic changes were observed at the pre-zoea stage. There was a synonymous SNP (G1593A) with a significantly different genotype frequency between the red and yellow populations (*p* < 0.05). The above results suggested that *scarb1* is involved in pigmentation by affecting the development of chromatophores.

## Simple Summary

This study investigates the role of scavenger receptor class B type I (*scarb1*) in the body color of *Neocaridina denticulata sinensis* (cherry shrimp). This research aims to understand the relationship between *scarb1* and pigmentation by analyzing the expression patterns of the *scarb1* gene in different color populations and developmental stages, performing RNA interference (RNAi) to silence *scarb1*, and identifying single nucleotide polymorphisms (SNPs) within the gene. The results showed significant differences in *scarb1* expression across color populations and developmental stages, with the highest expression in red shrimp and the pre-nauplius stage. Silencing *scarb1* via RNAi increased chromatophore development in the metanauplius stage. A specific SNP in *scarb1* showed significantly different frequencies between yellow shrimp and shrimp of other colors. This study concludes that *scarb1* plays a role in cherry shrimp pigmentation by influencing chromatophore development. This research provides insights into the genetic mechanisms underlying body color in cherry shrimp, which can be utilized to improve breeding strategies for desired color variations, enhancing the shrimp’s market value and benefiting the ornamental aquaculture industry.

## 1. Introduction

Cherry shrimp (*Neocaridina denticulata sinensis*) is a small shrimp that is widely distributed in the freshwater waters of Asia [1]. This species has strong environmental adaptability, with a wide tolerance range of temperatures and pH levels [2]. Red or blue mutants often appear in natural populations, in which almost all individuals are wild-type with some black spots. Since the 1990s, red mutants have been cultivated and have gradually become an ornamental shrimp named cherry shrimp [3]. Red cherry shrimps stand out from the green background of aquatic plants and have become a major fresh ornamental shrimp since 2003 [4]. Mutants of other colors were also selected from natural populations according to the cultivation process of red mutants. Populations with various body color phenotypes have been gradually cultivated through multiple generations of intragroup mating. Red, yellow, and blue populations are the most common ornamental shrimp of *N. d. sinensis*. Carotenoids are the main cause of color in most crustaceans [5]. Our previous data suggested that the color of cherry shrimp was mainly from various carotenoids.

Carotenoids, a type of natural pigment with the widest distribution, are found in a variety of organisms, including photosynthetic bacteria, archaea, fungi, algae, plants, and animals [6,7]. Almost all animals cannot synthesize carotenoids de novo, but obtain and convert carotenoids from food [8]. Initially, it was posited that carotenoids undergo transmembrane transport via simple diffusion [9]; however, subsequent discoveries indicating the existence of selectivity and saturation have substantiated that facilitated diffusion is the principal mode of absorption [10]. The first identified transporter of carotenoids was *scarb1*, through which intestinal epithelial cells absorb carotenoids from intestinal contents. Following the knockout of *scarb1* in mice, the absorption of carotene and carotenol in the intestinal tenue significantly decreased [11]. In addition, cluster determinant 36 (*CD36*) has also been proven to be another transporter of carotenoids, involved in the transmembrane transport of carotenoids in intestinal epithelial cells [12]. Both *scarb1* and *CD36* are transmembrane glycoproteins with a large extracellular domain. Their 3D structures, obtained from homologous modeling, indicate that the large cavity throughout the entire molecule might serve as a channel for transporting lipids [13,14]. The two proteins recognize their ligands using molecular patterns rather than specific epitopes, resulting in a variety of ligand types [15]. *Scarb1* also transports vitamin E and vitamin K besides carotenoids [16,17]. By binding to high-density lipoproteins (HDLs), scarb1 plays an important role in cholesterol transmembrane transport [18]. There are more ligands of CD36, including carotenoids, long-chain lipids, lipoproteins, thrombospondin-1, collagen, apoptotic cells, amyloid B, and red blood cells infected with malaria [18].

The function of *scarb1* in carotenoid transportation is highly conserved in evolution. In *Drosophila*, the *scarb1* homolog *ninaD* transports zeaxanthin and β-carotene, and the loss of ninaD function is associated with the loss of compound eye pigments [19]. A non-synonymous mutation of *ninaD* leads to a lack of carotenoids, retinoic acid, and vitamin E in *Drosophila*. *NinaD* expression in the midgut of *Drosophila* larvae is necessary for the occurrence of compound eyes [20]. The silkworm homolog of *scarb1* is a key factor in the accumulation of carotenoids in silk, and loss-of-function mutations can produce a phenotype of white cocoons [18]. The color of feathers and skin in canaries (*Serinus canaria*) also requires *scarb1*, which mutates and inactivates to produce a phenotype of recessive white feathers [21]. Moreover, there is an extremely low level of carotenoids in the blood and tissues of the white mutant, an organism that is severely deficient in vitamin A [21].

There were different expression levels of *scarb1* in our previous comparison of the transcriptomes of various colored populations of cherry shrimp [22]. In this paper, we obtained the expression profiles of *scarb1* in different color populations and developmental stages using qPCR. By silencing *scarb1* via RNAi to corroborate its function in the embryos, a SNP in *scarb1* was identified and its correlation analysis with color populations was processed. Our data could provide a new perspective for the functional analysis of the *scarb1* gene in cherry shrimp.

## 2. Materials and Methods

### 2.1. Experimental Animals

We used four different color populations of cherry shrimp during the experiment: red, yellow, blue, and wild populations. All color populations have undergone more than three years of breeding and have pure colors and a stable genetic inheritance. Thirty adult females and 10 males were cultured in a glass tank (40 cm × 30 cm × 25 cm) containing 500 g aqua soil and two 5 cm segments of *Elodea nuttallii*. The cultivation medium was tap water that had been aerated for more than 24 h, with half of the water changed every three days. The water temperature was maintained at 25 ± 1 °C. All shrimp were transferred to a new glass tank once a month. After three months, offspring grew up to about 1.2 cm in length, with about 300 individuals.

The cherry shrimp were frozen in liquid nitrogen and crushed in a mortar cooled by liquid nitrogen. The fine powder of the samples served as a biological replicate and was stored at −80 °C. There were seven biological replicates for each color population. For sampling the tissues of cherry shrimp, seven offspring were removed from a culture tank to a small breaker with 30 mL water on ice. After low-temperature paralysis, an individual was placed under a dissecting microscope to dissect and collect tissues from its compound eyes, hepatopancreas, muscles, epidermis, and digestive tract. All samples were firstly frozen in liquid nitrogen and stored at −80 °C. For sampling cherry shrimp embryos, berried females were collected and transferred to a new glass tank. The development stage was identified by microscopic observation with an anatomic microscope, according to the description of Lu et al. [23]. The chromatophores first appear at the metanauplius stage and gradually mature through the pre-zoea and membrane-zoea stages [24]. Larvae from these three stages, as well as the pre-nauplius and post-larva stages, were selected for the detection of gene expression. Embryos were removed from female appendages using tweezers. All embryos of a berried female served as a biological replicate. There were seven replicates in each sampling stage for qPCR. All replicates were frozen in liquid nitrogen and stored at −80 °C.

### 2.2. Total RNA Extraction, cDNA Preparation, and Quantitative PCR (qPCR)

Total RNA extraction was processed with RNA-solv reagent (Omega Bio-tek, Inc., Norcross, GA, USA) according to the manufacturer’s protocol. Approximately 1 μg total RNA was used as a template to synthesize the first strand of its DNA. The cDNA synthesis reagent kit was from TIANGEN (Tiangen Co., Ltd., Beijing, China). The detailed operation process was carried out according to its manual. Primers (Table 1) for qPCR were designed using Primer3 online software (https://sourceforge.net/projects/primer3/, accessed on 17 March 2025) and synthesized by TsingKe Biotechnology Co., Ltd. (Beijing, China). The relative expression level of the target gene was calculated using the 2^−ΔΔCt^ method based on the internal reference gene of glyceraldehyde-3-phosphate dehydrogenase (*GAPDH*, GenBank accession MZ734609). The GenBank accession for *scarb1* is PRJNA1209655. The qPCR reaction was performed in a 10 μL reaction volume, including 0.25 μL of the forward primer (10 mM), 0.25 μL of the reverse primer (10 mM), 5 μL of SYBR green master mix (GOY-P2028, Coybio, Shanghai, China), and 4.5 μL of the cDNA template. PCR was carried out on a QuantStudio 6 Flex instrument (Applied Biosystems, Carlsbad, CA, USA), with the following procedure: 95 °C for 5 min, 40 cycles of 95 °C for 10 s, 60 °C for 10 s, and 72 °C for 10 s.

### 2.3. dsRNA Synthesis and RNAi

In vitro transcription was used to prepare the dsRNA for RNAi. We chose an approximately 300 bp sequence located at the 5′ end of the open reading frame (ORF) as the template for synthesizing dsRNA in vitro. The PCR primers for target DNA amplification are shown in Table 1. The details of the dsRNA synthesis were described previously [25]. In brief, the target DNA fragment was added to the T7 promoter sequence at the 5′ end or 3′ end, respectively, by the second PCR amplification, which added the T7F primer or T7R primer (Table 1), which was added to the T7 promoter sequence at the 5′ end of the specific primer. The new DNA fragment with the T7 promoter sequence was chosen as the template for the sense ssRNA or anti-sense ssRNA of the target gene. The sense and anti-sense ssRNA strands were equivalently mixed and annealed to the dsRNA. The annealing procedure was set at 75 °C for 15 min, 65 °C for 15 min, and then dropped to 25 °C at a rate of 0.2 °C/s. The dsRNA of *EGFP* served as a control to verify the effect of non-specific dsRNA. All primers used in dsRNA preparation are listed in Table 1.

The embryos of the expected stages were collected as above. All embryos from the same berried shrimp were rinsed three times with ultrapure water (0.01 μm) and divided into two equal parts. An appropriate amount of dsRNA was added to ensure that the final concentration was 5 μg/mL. One part was exposed to 5 μg/mL *scarb1* dsRNA for 24 h as the treated group (TG), and the other was exposed to 5 μg/mL *EGFP* dsRNA for 24 h as the control group (CG). After 24 h post-transfection, the samples from the TG and CG were collected for further qPCR analyses and observation. Embryos from one female served as one biological replicate. There were five biological replicates for each gene interference. After dsRNA exposure, the embryonic phenotype was observed and photographed under a microscope.

The red pixel brightness ratio of chromatophores was obtained using Adobe Photoshop2020 software. In general, chromatophores were selected using the magnetic lasso tool, and the histogram of the selected area showed the red pixel brightness and total pixel brightness. The red pixel brightness (RPB) ratio was defined as the ratio of red pixel brightness to total pixel brightness. The length and width values of chromatophore clusters in compound eyes were calculated using Image J1. The state of pigment distribution within chromatophores was assessed by a 5-point scale as follows: stage 1, maximal concentration; stage 5, maximal dispersion; and stages 2, 3, and 4, intermediate conditions, as defined by Hogben and Slome [26]. The pigment distribution scale (PDS) of an embryo was presented by the mean scale of all of its chromatophores.

### 2.4. Calling and Filtrating SNPs

The calling of SNPs was processed using the Genome Analysis ToolKit (GATK) (v4.5.0.0) [27,28], based on our previous transcriptome data from 216 shrimp from four color populations (red, yellow, blue, and wild) [29]. The potential SNPs were filtered by quality scores ≥ 30, reading depths ≥ 10, and minor allele frequencies ≥ 0.05 to select high-quality SNPs [30]. The frequency of mutant (minor) alleles on each locus of each population was calculated using the read counts of mutant alleles divided by all read counts on the locus. The frequency of the reference allele equaled 1 minus the frequency of mutant alleles. Subsequently, the heterozygosity (*H*) of each population was calculated using the formula: *H* = 2 × reference allele frequency × mutational allele frequency, and the average heterozygosity of all populations (*H_S_*) was defined as the average value of *H* in all populations. Moreover, the total frequency of the reference allele and mutational allele on each locus was calculated using the total allelic read counts divided by all read counts on the mono locus of the total individuals, and the total heterozygosity (*H_T_*) was calculated by the formula: *H_T_* = 2 × total reference allele frequency × total mutational allele frequency. Finally, the *F_st_* value was estimated by the formula *F_st_* = (*H_T_* − *H_S_*)/*H_T_*.

### 2.5. Genotyping of Candidate SNPs

SNPs ranking in the top 5% according to *F_st_* value were defined as candidate SNPs. Genotyping of a candidate SNP in *scarb1* was conducted using GT-seq (genotyping-in-thousands by sequencing). GT-seq is a method that uses next-generation sequencing of multiplexed PCR products to generate genotypes from targeted SNPs for thousands of individuals in a single Illumina HiSeq lane [31]. The details of GT-seq were described previously [30].

The individuals of four color populations were used for genotyping. There were 384 individuals in each population. A pleopod of a shrimp was used to extract genomic DNA using Chelex-100 (Solarbio Science & Technology Co., Ltd. Beijing, China). Pearson’s χ^2^-test was performed to investigate the relationships between the SNP genotype and body color. *p* < 0.05 was considered significant.

The numbers of codon usage of an amino acid were counted using codon W software (v1.4.2) based on the coding sequence (CDS) of cherry shrimp transcriptomes. Relative synonymous codon usage (RSCU) and synonymous codon frequency (SCF) were used to represent codon usage bias. RSCU represents the ratio between the usage count of a synonymous codon and its expected occurrence. The expected value of RSCU is 1, which is more than the mean of preferable usage. SCF denotes the frequency of codons encoding the same amino acid, with a value closer to 1 indicating a stronger preference. RNA secondary structures were predicted using the mfold of UNAFold (http://www.unafold.org/mfold/applications/rna-folding-form-v2.php, accessed on 17 March 2025). The multiple sequence alignment of scarb1 homologs was analyzed using Clustal W2.1 and edited using BioEdit 7.0.

## 3. Results

### 3.1. Scarb1 Expression Profiles

**Figure 1:**
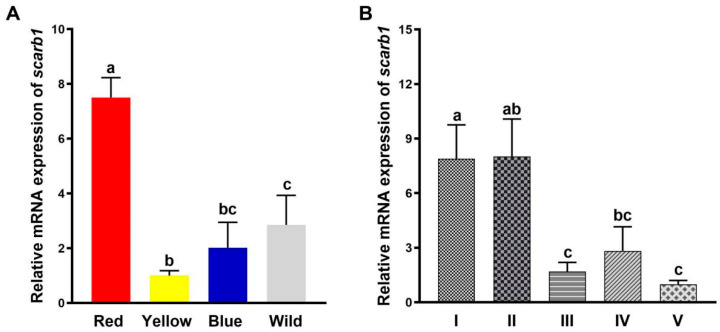
Relative expression of *scarb1* in *Neocaridina denticulata sinensis*. (**A**) The relative expression levels of *scarb1* in different populations; (**B**) The relative expression levels of *scarb1* in different developmental stages. I: pre-nauplius stage; II: metanauplius stage; III: pre-zoea stage; IV: membrane-zoea stage; V: post-larva stage. Different letters indicate significant differences (*p* < 0.05). Error bars represent the mean ± standard error.

There were significant differences in *scarb1* expression between red, blue, yellow, and wild populations (*p* < 0.05) (Figure 1A). The expression level in the red population was significantly higher than in the other three populations (*p* < 0.05), and the expression in the wild population was significantly higher than in the yellow population (*p* < 0.05). However, there was no significant difference between wild and blue populations (*p* > 0.05) (Figure 1A).

*Scarb1* expression levels at the nauplius stage were significantly higher than at the zoea stages (*p* < 0.05) (Figure 1B). There was no significant difference in the red pixel brightness (RPB) ratio and the pigment distribution scale (PDS) between pre-nauplius and metanauplius groups and pre-zoea, membrane-zoea, and post-larva groups (*p* > 0.05) (Figure 1B).

### 3.2. Functional Analyses of Scarb1 via RNAi

**Figure 2:**
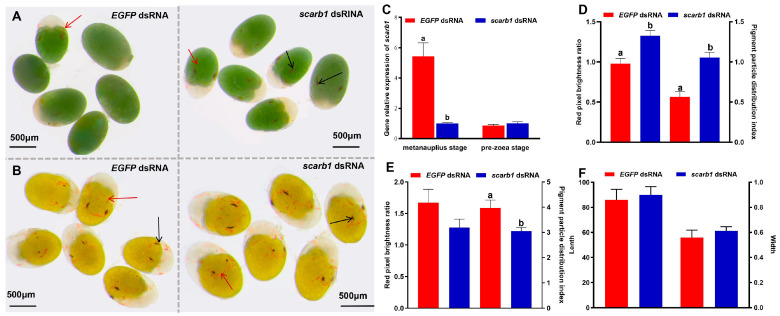
The effect of chromatophores after *scarb1* interference in *Neocaridina denticulata sinensis* embryos. (**A**) The metanauplius stage after *scarb1* interference. *EGFP* dsRNA was the non-specific control and *scarb1* dsRNA was the *scarb1* interference group; (**B**) The pre-zoea stage after *scarb1* interference. *EGFP* dsRNA was the non-specific control and *scarb1* dsRNA was the *scarb1* interference group; (**C**) The relative expression level of *scarb1* in metanauplius and pre-zoea stages after RNAi; (**D**) The red pixel brightness (RPB) ratio and the pigment distribution scale (PDS) of the metanauplius stage; (**E**) The red pixel brightness (RPB) ratio and the pigment distribution scale (PDS) of the pre-zoea stage; (**F**) The length and width of chromatophore clusters in compound eyes. The black arrow indicates a chromatophore cluster in compound eyes, and the red arrow indicates an erythrophore. Different lowercase letters indicate significant differences (*p* < 0.05). Error bars represent the mean ± standard error.

There appeared to be phenotypic changes in chromatophores (Figure 2A). At the metanauplius stage, the number of chromatophores in the treatment group was more than in the control group, especially near the ommateum (Figure 2A). There appeared to be two clusters of ommateum chromatophores and several developed chromatophores with branches in the treatment group (Figure 2A). On the other hand, only one individual in the control group had a cluster of ommateum chromatophores, and most individuals had one pair of chromatophores without any branching (Figure 2A). Meanwhile, the treatment of *scarb1* dsRNA exposure significantly decreased its expression (*p* < 0.05) at the metanauplius stage (Figure 2C). The PDS and RPB ratios of the treatment group were significantly higher than those of the control group (*p* < 0.05) (Figure 2D).

At the pre-zoea stage, dsRNA exposure had no effect on silencing *scarb1* expression levels, with no significant difference between the treatment group and the control group (Figure 2B). There was no difference in the RPB ratio between the treatment group and the control group at the pre-zoea stage (*p* > 0.05) (Figure 2E). The number of chromatophores in the control group was similar to the treatment group (*p* > 0.05), but the PDS of the control group was higher than the treatment group (*p* < 0.05) (Figure 2E). The length and width of ommateum chromatophore clusters in the treatment group were higher than in the control group, but there was no difference between the two groups (*p* > 0.05) (Figure 2F).

### 3.3. SNP Genotyping

**Figure 3:**
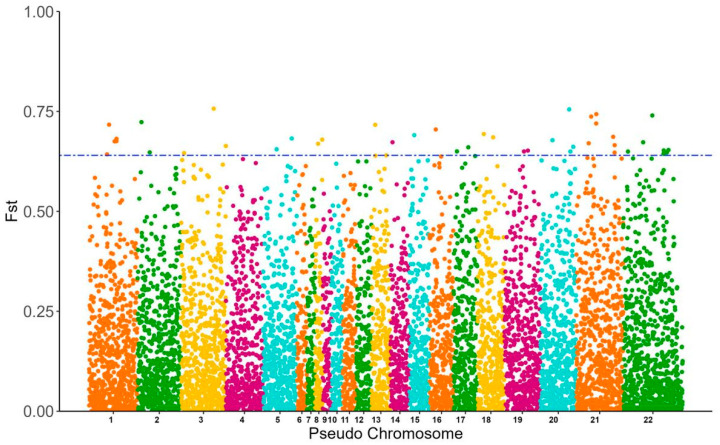
*Fst* of high-quality SNPs in three color populations (red, yellow, and wild). All unigenes of the transcriptome were divided into 22 equal parts based on their ID number as 22 pseudochromosomes. The blue dashed line represents the lowest *Fst* in the top 5% of SNPs.

**Figure 4:**
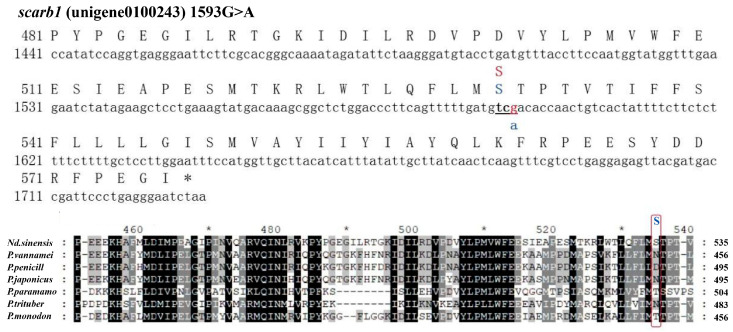
Partial sequence of *scarb1*. Capital letters represent amino acids, lowercase letters represent nucleotides, and underlining represents triplet codons. The red box shows the amino acids coded by SNP 1593 G>A in cherry shrimp and other species, and the upper blue letter of the box represents the amino acid of cherry shrimp after mutation. A black background indicates that the amino acid at this location is identical in all comparison species, and a gray background indicates that the amino acid at this location is similar in all comparison species.

**Figure 5:**
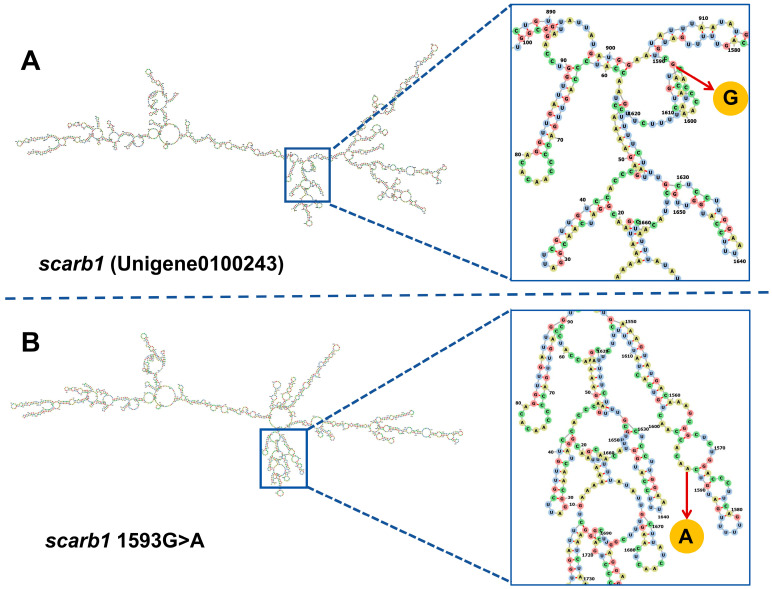
Effect of the SNP on RNA secondary structure. The black lines are RNA secondary structures, the color diagram is the enlargement of the corresponding box part, and the arrow points to the exact position of the SNP. (**A**) The original RNA structure prediction of *scarb1*; (**B**) The influence of the 1593G>A change on the secondary structure of RNA.

**Figure 6:**
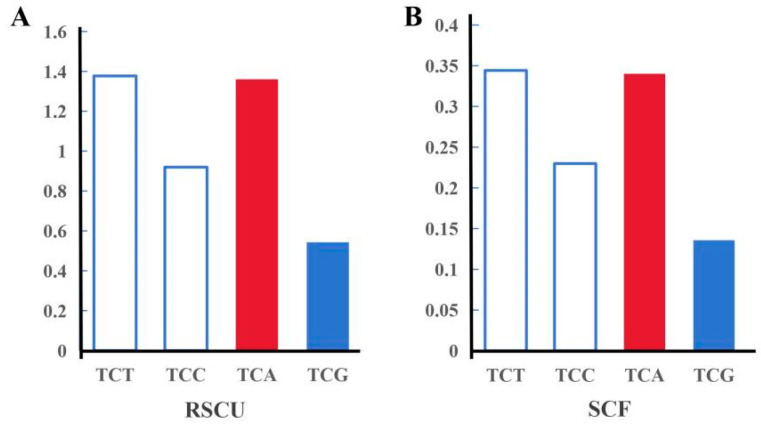
Codon usage of serine in *Neocaridina denticulata sinensis*. (**A**) RSCU stands for Synonymous codon usage; (**B**) SCF stands for synonymous codon frequency.

There were 326,902 potential SNPs, as called by GATK, and 8424 high-quality SNPs after filtration (Figure 3). The average *F_st_* of high-quality SNPs was 0.164, and the *F_st_* of the top 5% was 0.640. There was one candidate SNP in *scarb1*, named G1593A, which was a synonymous mutation and coded for serine (S521). The SNP was located at the third position of the serine codon (Figure 4). The second structure of the reference allele was different than that of the alternative allele (Figure 5). The TCG and TCA codons were from the reference allele and the alternative allele, respectively. The RSCU of TCG was 0.54 and was lower than that of TCA (1.36). The codon usage for the SNP with TCG changed to TCA changed from 13.5% to 34.0%. The other two codons of serine were TCT and TCC, with RSCU values of 1.38 and 0.92 (Figure 6).

The genotype of all red individuals was AA, and the genotype of yellow individuals was GA (Table 2). There was a significant genotype frequency between the red population and the yellow population (*p* < 0.05). Meanwhile, the blue population and the wild population carried two genotypes (AA and GA), with no significant genotype frequency in the two populations (*p* > 0.05). Most individuals in the blue population and the wild population were AA homozygotes, with a few individuals being GA heterozygotes. The genotype frequency of the yellow population was significantly different from the blue population and the wild population (*p* < 0.05).

## 4. Discussion

Scavenger receptors are a kind of cell surface protein with the ability to clear non-self substances or self mutated molecules and play a role in phagocytosis, endocytosis, adhesion, and signal transduction [32]. There are a total of 12 types of scavenger receptors, and their structures vary greatly. Scavenger receptor class B (*scarb1*) has two transmembrane domains, one large extracellular domain, and two small intracellular domains. *Scarb1* selectively transports lipids and is a major cell membrane surface receptor for HDL, which was found to be involved in the uptake and transport of carotenoids [33].

Scarb1 is a multifunctional protein in animals that participates in physiological processes. It has recently been reported that scarb may be an important mediator of astaxanthin uptake in shrimp [34]. A knockout of or decrease in the expression of *scarb1* will lead to changes in carotenoid content in various animals, such as the sharp decrease in the absorption of β-carotenoid and zeaxanthin in the retinal pigment cells of mammals [11,35], the lack of carotenoids in canary feathers [21], the decrease in astaxanthin content and the appearance of white skin in carp [36], impaired carotenoid pigment deposition function in zebrafish [37], and the lack of carotenoids and retinoids in *Drosophila* [38,39]. The overexpression of *scarb1* in mammalian retinal pigment epithelial cells led to greater carotenoid absorption [10]. The higher expression of *scarb1* was consistent with the appearance of numerous chromatophores at the pre-nauplius stage of cherry shrimp. The results suggested that *scarb1* was involved in the transport of chromatophores and pigmentation in chromatophores. After silencing *scarb1* in cherry shrimp, the development of chromatophores and pigmentation was inhibited. The result was consistent with the above articles, which suggested that scarb1 might promote the absorption of carotenoids in chromatophores. In addition, *scarb1* expression did not match any kind of carotenoid content, except β-cryptoxanthin, in the four color populations (blue, red, yellow, and wild) of cherry shrimp. Because of the diversity of scarb1 ligands, scarb1 in cherry shrimp might prefer to transport β-cryptoxanthin more than the other carotenoids. The RNAi subject was erythrophores. In cherry shrimp embryos, erythrophores are the only chromatophores that mainly contain astaxanthin [25]. The decrease in β-cryptoxanthin transport might had a minor effect on the hue of embryo erythrophores, so the effect of *scarb1* RNAi in cherry shrimp was not as obvious as in the above research. However, our previous data showed that once pigmentation has occurred in erythrophores, the color phenotype almost never changes, and RNAi also had no effect. In this paper, RNAi treatment was applied at the beginning of the occurrence of erythrophores (the metanauplius stage) for a greater effect on color phenotype. Once a large number of erythrophores appeared at the pre-zoea stage, especially when clusters of ommateum chromatophores became thicker, it became difficult for RNAi to alter the color phenotype or even to reduce the expression level of the target genes. There is only 1–2 h for dsRNA exposure at the pre-zoea stage. The asynchronous development of cherry shrimp is a common event. It is difficult to obtain embryos from a maternal parent that has developed synchronously for RNAi. Therefore, this paper did not achieve the expected result of RNAi at pre-zoea. The precise reason for the non-functioning dsRNA exposure remains unknown to us. The inability of dsRNA to enter mature pigment cells could be an important factor.

G1593A is a synonymous mutation, and its encoding amino acids are conserved. The genotyping of G1593A showed that the SNP was associated with body color. Heterozygous GA was the major genotype in the yellow population, and homozygous AA was the major genotype in the other three populations. Traditionally, a synonymous mutation does not cause a change in amino acids and, therefore, does not affect protein structure and function. However, recent studies have shown that synonymous SNPs can affect protein expression and function [40,41,42]. If a synonymous SNP is in linkage disequilibrium with other common functional non-synonymous polymorphisms, the non-synonymous SNP will reflect the effect of the non-synonymous SNP. For example, rs5888, a synonymous SNP in exon 8 of human *scarb1*, is close to two non-synonymous SNPs [43,44,45]. A synonymous SNP in exon 26 (C3435T) of human P-gp is in linkage disequilibrium with other functional non-synonymous polymorphisms, such as G2677T [46]. Our SNP calling results based on the transcriptomes of cherry shrimp showed that there were other non-synonymous SNPs in the exons of *scarb1* of cherry shrimp.

The second possible explanation of synonymous SNP function is that allele-specific differences in mRNA folding could influence splicing, processing, or translational control and regulation. The rs5888 variant has been proven to affect *scarb1* RNA’s secondary structure and protein translation and is significantly associated with reduced scarb1 protein expression and function [43]. The predicted results of mRNA showed that G1593A changed the secondary structure of *scarb1*. The different mRNA folding of the alternative allele could alter mRNA splicing or protein translation, ultimately eliciting changes at the protein level. Subsequent research will analyze the protein level to infer whether it affects the translation process and subsequently affects the coloring process.

A third explanation is that the use of rare codons appears to influence the translation rate, which in turn affects protein folding [47]. G1593A is located in the third base of the codon with the largest effect on protein folding. The different frequencies of genotypes might indicate that there were various protein conformations in the four color populations. In general, tRNA corresponding to high-usage codons has a high abundance [48]. Natural selection may be one of the reasons for forming this correspondence [49,50]. According to Alphafold, S521 was located in the C-terminal transmembrane domain of cherry shrimp *scarb1*. In human *scarb1*, the homo-oligomerization is driven by interactions between C-terminal transmembrane domains, in which no non-synonymous polymorphism is found. The translation rate of the C-terminal transmembrane domain might have an influence on its conformation and the function of the whole protein.

The SNPs of human *scarb1* are associated with the concentration of β-carotene in plasma, explaining 50% of the concentration variance [51] and suggesting that *scarb1* may be one of the major genes influencing plasma β-carotene levels. Our genotyping results also suggested that *scarb1* plays a role in body color, especially in the yellow color, of cherry shrimp. An animal’s body color is a quantitative trait controlled by many genes. Certain genes may exert a substantial influence on body coloration, while others may have little influence. The inheritance of human eye color serves as an excellent example [52], where SNPs within eight major genes can be utilized to predict the iris’s color [53]. The genotype of G1593A suggested that *scarb1* might be one of the major genes in the yellow phenotype. Interestingly, there is no genotype difference between the blue and wild populations, which may be regulated by another factor, for example, non-coding RNA (ncRNA). Findings have revealed that *Apis mellifera* could change the body color, perhaps by altering the expression of ncRNA-related key genes [54]. This indicates that G1593A of *scarb1* might be one of the major genes responsible for the yellow phenotype, but within the blue population, it may be regulated by more factors.

## 5. Conclusions

In summary, the *scarb1* expression profiles of embryos and populations showed that its expression level had a close relationship with chromatophore occurrence and body color. Although reducing the expression of *scarb1* by RNAi affected pigmentation and pigment distribution in erythrophores, its influence on erythrophore phenotype was limited. Conversely, the G1593A genotype of the yellow population significantly deviated from other body colors; this result suggested a substantial influence of *scarb1* on xanthophores, which are the major chromatophores of a yellow individual. Unfortunately, we are unable to validate this hypothesis by RNAi during the occurrence of xanthophores, which first appear at the post-larval stage. The dsRNA could not be delivered into post-larvae by soaking, while injections led to a high mortality rate in post-larvae. In the future, gene knockout may be used to study *scarb1*’s role in the occurrence of xanthophores.

## Figures and Tables

**Table 1:** The PCR primers used in this study.

Primer Name	Primer Sequences (5′–3′)	Application
scarb1-Fq	ATGGGAGCTCCAGCCTTTAT	qPCR
scarb1-Rq	GCATGCTTTTCCTCTTCAGG	qPCR
scarb1-Fd	CGTTGTCCACCCAGAGAAAA	dsRNA generation
scarb1-Rd	CGTTGTCCACCCAGAGAAAA	dsRNA generation
scarb1-F1	CCTTCTCTTGAGGATGCCACT	Target sequence amplification
scarb1-R1	TCCTTGAACAGCCTCTCGTT	Target sequence amplification
scarb1-F2	CGACAGGTTCAGAGTTCTACAGTCCGACGATCCCTTCTCTTGAGGATGCCACT	Add adapter
scarb1-R2	GCTCGTCGTGACGCCATGACGTCCTTGAACAGCCTCTCGTT	Add adapter
ds-EGFP F	GGTGAACTTCAAGATCCGCC	dsRNA generation
ds-EGFP R	CTTGTACAGCTCGTCCATGC	dsRNA generation
GADPH-Fq	CGGTGCTGCTCAGAATATCA	qPCR
GADPH-Rq	TTACCAAGGCGAACGGTAAG	qPCR
T7F	TAATACGACTCACTATAGGG	Adapter
T7R	CCCTATAGTGAGTCGTATTA	Adapter

**Table 2:** Genotyping of different color populations.

Population		Red	Yellow	Blue	Wild
Genotype	AA	345 ^a^	0 ^b^	329 ^a^	331 ^b^
	GA	0	345	16	14
Allele	A	1.000 ^a^	0.500 ^b^	0.977 ^a^	0.980 ^a^
	G	0.000	0.500	0.023	0.020

The ‘genotype’ row represents the number of individuals, the ‘allele’ row represents the rate. Superscript letters of numbers indicate the significance level of a line. The same letter represents no significance (*p* > 0.05), while different letters represent significance (*p* < 0.05).

## Data Availability

All data used in this study are available upon request from the corresponding author.
